# Impact of Gold-Standard Label Errors on Evaluating Performance of Deep Learning Models in Diabetic Retinopathy Screening: Nationwide Real-World Validation Study

**DOI:** 10.2196/52506

**Published:** 2024-08-14

**Authors:** Yueye Wang, Xiaotong Han, Cong Li, Lixia Luo, Qiuxia Yin, Jian Zhang, Guankai Peng, Danli Shi, Mingguang He

**Affiliations:** 1 State Key Laboratory of Ophthalmology, Zhongshan Ophthalmic Center, Sun Yat-sen University, Guangdong Provincial Key Laboratory of Ophthalmology and Visual Science, Guangdong Provincial Clinical Research Center for Ocular Diseases Guangzhou China; 2 School of Optometry The Hong Kong Polytechnic University Kowloon China (Hong Kong); 3 Guangzhou Vision Tech Medical Technology Co, Ltd Guangzhou China; 4 Research Centre for SHARP Vision The Hong Kong Polytechnic University Kowloon China (Hong Kong); 5 Centre for Eye and Vision Research Hong Kong China (Hong Kong)

**Keywords:** artificial intelligence, diabetic retinopathy, diabetes, real world, deep learning

## Abstract

**Background:**

For medical artificial intelligence (AI) training and validation, human expert labels are considered the gold standard that represents the correct answers or desired outputs for a given data set. These labels serve as a reference or benchmark against which the model’s predictions are compared.

**Objective:**

This study aimed to assess the accuracy of a custom deep learning (DL) algorithm on classifying diabetic retinopathy (DR) and further demonstrate how label errors may contribute to this assessment in a nationwide DR-screening program.

**Methods:**

Fundus photographs from the Lifeline Express, a nationwide DR-screening program, were analyzed to identify the presence of referable DR using both (1) manual grading by National Health Service England–certificated graders and (2) a DL-based DR-screening algorithm with validated good lab performance. To assess the accuracy of labels, a random sample of images with disagreement between the DL algorithm and the labels was adjudicated by ophthalmologists who were masked to the previous grading results. The error rates of labels in this sample were then used to correct the number of negative and positive cases in the entire data set, serving as postcorrection labels. The DL algorithm’s performance was evaluated against both pre- and postcorrection labels.

**Results:**

The analysis included 736,083 images from 237,824 participants. The DL algorithm exhibited a gap between the real-world performance and the lab-reported performance in this nationwide data set, with a sensitivity increase of 12.5% (from 79.6% to 92.5%, *P*<.001) and a specificity increase of 6.9% (from 91.6% to 98.5%, *P*<.001). In the random sample, 63.6% (560/880) of negative images and 5.2% (140/2710) of positive images were misclassified in the precorrection human labels. High myopia was the primary reason for misclassifying non-DR images as referable DR images, while laser spots were predominantly responsible for misclassified referable cases. The estimated label error rate for the entire data set was 1.2%. The label correction was estimated to bring about a 12.5% enhancement in the estimated sensitivity of the DL algorithm (*P*<.001).

**Conclusions:**

Label errors based on human image grading, although in a small percentage, can significantly affect the performance evaluation of DL algorithms in real-world DR screening.

## Introduction

In recent years, the application of artificial intelligence (AI) in identifying ocular lesions and diseases has gained increasing popularity [1-4]. One prominent example is the application of deep learning (DL) in fundus image–based diabetic retinopathy (DR) screening [5-8]. Despite the widely reported high accuracy in existing studies, as well as the advantages in efficiency and cost-effectiveness, the real-world implementation of DL-based DR-screening models still faces challenges [9]. A major concern is the gap observed between the high laboratory performance and the unsatisfying real-world performance. For example, although most DL algorithms have achieved a sensitivity of over 90% in previous research, the reported sensitivity ranges from 55% to 85% in real-world tasks [4,7,10]. A better understanding of the reasons for the performance gap so as to enhance the real-world performance of DL-based models could help promote the implementation of AI on the ground, but related evidence is scarce.

In the development of medical AI, human expert labels usually serve as the gold standard to compare with and evaluate model results [11]. The accuracy of AI model–based tasks critically depends on high-quality labels. Assembling large data sets with human expert labels can be a huge task, and ensuring the quality of the gold standard remains an open problem [12]. In real-world image data sets, the gold standard is usually generated by trained graders who manually assign grades to the data [13-15]. However, human error is inevitable in this process, leading to problems, such as bias, subjective judgement, inadequate repeatability, and consistency. Studies have reported significant discrepancies in DR grading among human graders, with sensitivities ranging from approximately 60% to 90% [16]. As a result, using manual grading outputs as an objective reference standard can be problematic when the accuracy of manual grading is in question [11,17,18].

We hypothesized that errors in human labels may exist in the real world and exert a nonnegligible impact on real-world DL model performance. Thus, in this study, we included over 0.7 million fundus images from 0.2 million individuals from a nationwide DR-screening program, based on which we aimed to assess the gap between lab-reported and real-world performance of a previously validated DL-based DR-screening model, identify the existence of label errors, and assess the impact of those label errors on the observed performance gaps.

## Methods

### Ethical Considerations

The study adhered to the tenets of the Declaration of Helsinki and received approval from the Institutional Review Board of the Zhongshan Ophthalmic Centre (2023KYPJ108) and the Chinese Foundation for Lifeline Express. All images were fully anonymized. Written informed consent was obtained from all Lifeline Express participants. Given the retrospective nature of this study using anonymized images, the ethics review board exempted this study from additional informed consent.

### Real-World Data Set

**Figure 1:**
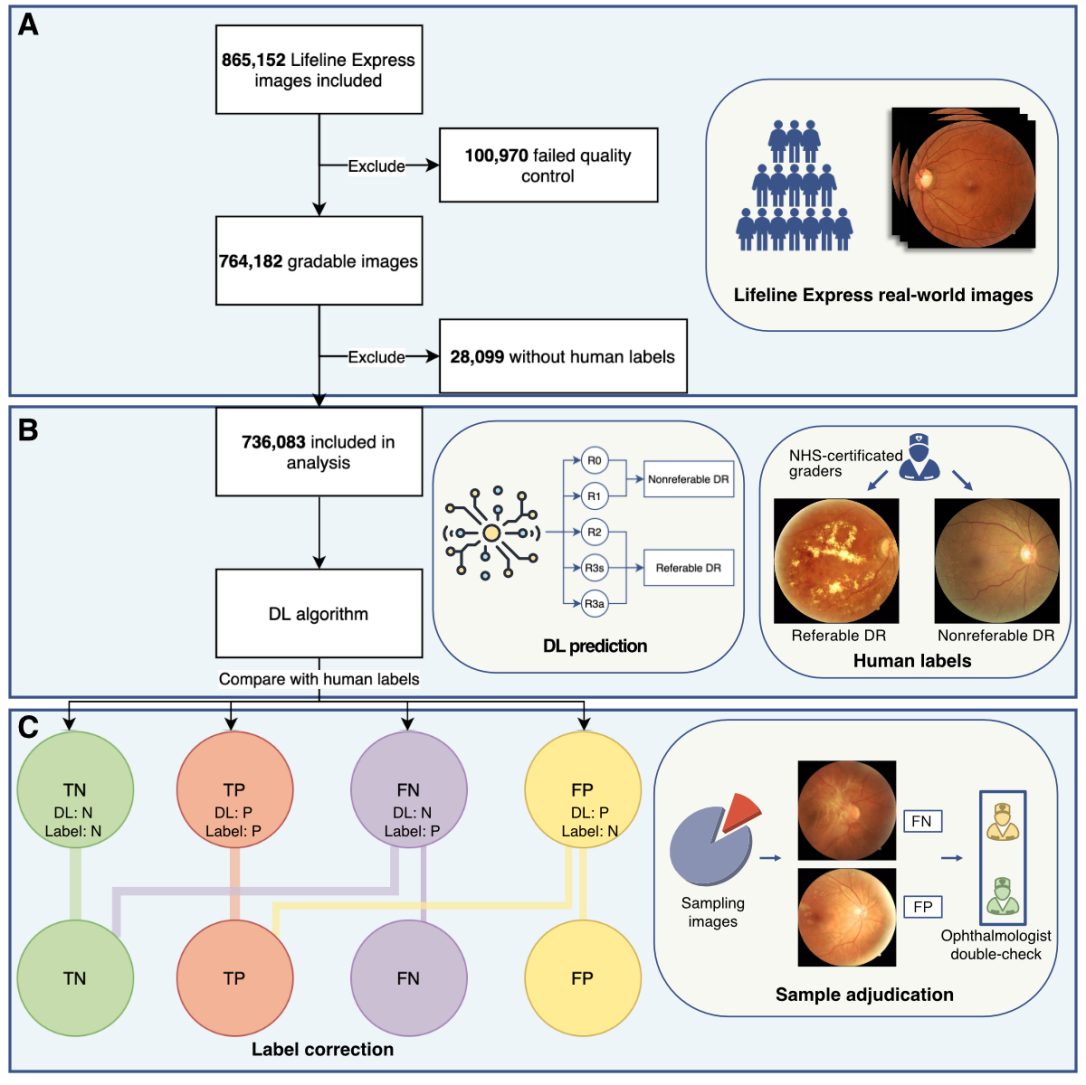
Schematic pipeline of the study design. DL: deep learning; DR: diabetic retinopathy; FN: false negative; FP: false positive; NHS: National Health Service; TN: true negative; TP true positive.

The pipeline of this study is depicted in Figure 1. The overall design of this study comprised 3 sections. First, more than 0.8 million images were collected from a real-world data set, and all images were included except those that failed quality control or lacked human labels. Second, fundus images were reviewed by National Health Service (NHS)–certificated human graders and manually classified as nonreferable DR (R0 and R1) or referable DR (R2, R3s, and R3a) according to NHS DR-screening guidelines. Next, a previously validated DL model was deployed for identifying referable DR, and the performance of the DL model was evaluated against human labels. Third, a random sample of images, comprising false-negative (FN) and false-positive (FP) subsets, was extracted for adjudication by 2 ophthalmologists. The details of the process are given next.

When adjudication yielded consistent results with DL instead of human labels, the errors of the labels were documented. The extracted sample’s error rates were used to recalculate the number of positive and negative cases in the entire data set, as postcorrection labels. The diagnostic performance of the DL algorithm was reassessed against the postcorrection labels.

We included 865,152 color fundus images of participants who participated in a nationwide real-world DR screening program, the Lifeline Express DR Screening Program, between 2014 and 2019. For each participant, nonmydriatic, 45° field color fundus images were captured using locally available imaging devices (including AFC-230, NIDEK; Canon CR-DGi; FundusVue, Cystalvue) with at least 1 image centered on the macula or optic disc. The size of the images was 512 × 512 pixels, at a resolution of 72 pixels per inch. Images were subsequently sent to 5 different central grading centers (Peking Union Medical College Hospital, Beijing Tongren Hospital, Peking University Third Hospital, Joint Shantou International Eye Center, and Zhongshan Ophthalmic Center) and graded for DR by human graders who possessed English NHS-certified qualifications. The following images were excluded: (1) those that failed the quality control procedure and (2) those without human labels. Details regarding the quality control and DR-grading criteria can be found in Table S1 in Multimedia Appendix 1.

### Grading Criteria

Based on NHS DR-screening guidelines, each fundus image was classified into 1 of 5 grades: R0 (no DR), R1 (background DR), R2 (preproliferative DR), R3s (static proliferative DR), or R3a (active proliferative DR). Referable DR was defined as preproliferative DR or worse (ie, R2, R3s, and R3a) and recorded as positive, whereas nonreferable DR (ie, R0 and R1) was recorded as negative. A participant was diagnosed as having referable DR if at least 1 of their gradable images was classified as positive.

The manual grading process involved the following stages. Initially, all images were assessed by a primary grader. Images classified as positive and a random sample of 15% of images classified as negative were then subjected to a second round of grading by another primary grader. Images with any discrepancy between the assessments of the 2 primary graders were referred to a secondary grader, whose conclusion served as a final determination. The resulting manual grading outputs formed human labels.

### Development of the DR-Grading DL Algorithm

The DR-grading algorithm is based on a convolutional neural network. The algorithm was trained using 71,043 retinal images from 36 ophthalmic departments and validated using an additional 106,244 retinal images from 3 population-based studies. Image preprocessing involved scaling image pixel values within a range of 0-1 and subtracting the local-space average color. Images were resized to 299 × 299 pixels. We performed data augmentation techniques on training data. This involved randomly shifting each image horizontally by 0-3 pixels and rotating it by 90°, 180°, or 270°.

The original version of the algorithm has been reported previously [19]. The original DL algorithm achieved an area under the receiver operating characteristic curve (AUROC) of 0.955 (sensitivity: 92.5%, specificity: 98.5%) in validation for the detection of referable DR. The algorithm used in our study was retrained using the Xception architecture to improve model performance, while using the same training data set. Max pooling of each Inception module and global average pooling were used in the Xception architecture. For training, a minibatch gradient descent size of 32 was used. The learning rate adopted an inverse time decay schedule with an initial value of 0.001.

### Adjudication and Human Label Correction

We adjudicated images that showed discrepancies between DL and human labels in referable DR classification, including FN (DL: negative, label: positive) and FP (DL: positive, label: negative). Adjudication was performed by 2 experienced ophthalmologists who were masked to the DL and human grading results; they used a back-to-back method in accordance with NHS DR-screening guidelines. Images with any discrepancy between the 2 ophthalmologists were sent to a senior ophthalmologist for reassessment, whose grading results were deemed as final.

Given the large number of images in this study, reviewing all images with discrepancies would have been impractical. Therefore, random samples were drawn for adjudication. To ascertain the representativeness of these random samples across the entire data set, a series of gradual samplings were conducted, varying in size from 100 to 500 FN images. Notably, a similar distribution of the estimated error rate was observed across all sample sizes (Figure S1 in Multimedia Appendix 1), suggesting that even a subset as small as 100 images can effectively represent the label distribution within the entire data set. Considering the total volume of FN and FP cases, we examined a 10% sample from FN cases and a 5% sample from FP cases, at both image and participant levels, aiming for a representative and manageable size of the sample. When adjudication yielded consistent results with DL instead of human labels, the label errors were documented in the FN and FP samples, respectively. Using the label error rates in the FN and FP samples, we recalculated the number of positive and negative cases in the entire data set, serving as postcorrection labels.

### Statistical Analysis

We evaluated the performance of the DL algorithm using pre- and postcorrection labels as the gold standard. Primary evaluations metrics for diagnostic performance included accuracy, precision, sensitivity (recall), specificity, and the *F*_1_-score. AUROC was calculated at the image level. These evaluations were conducted at both image and individual levels.

Label error rates in adjudicated samples were calculated as follows:

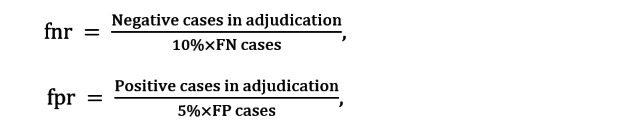



where fnr and fpr represent label error rates in FN and FP samples, respectively. Label changes during label correction included:

pn = fnr × FN cases,

np = fpr × FP cases,

where pn is the number of labels changing from positive to negative, while np is the number of labels changing from negative to positive. The total positive and negative cases after label correction were calculated as follows:

Total positive cases = Positive labels + np – pn

Total negative cases = Negative labels + pn – np

Comparisons of diagnostic performance were conducted using chi-square tests. Adjudication was conducted on the open source LabelMe platform [20]. All statistical analyses were performed using R version 4.1.2 (Foundation for Statistical Computing). A 2-sided *P* value of <.05 was considered statistically significant.

## Results

### Precorrection Model Performance

Of the 865,152 images acquired from 251,535 participants, 736,083 (85.1%) images from 237,824 (94.5%) participants were included in this analysis. Table 1 shows the results of the DL algorithm and precorrection labels for detecting referable DR. At the image level, DL-based grading yielded 9339 (1.3%) FN, 57,886 (7.9%) FP, 632,457 (85.9%) true negative (TN), and 36,401 (5.0%) true positive (TP) images against precorrection labels. The DL algorithm yielded an AUROC of 0.927, a sensitivity (recall) of 79.6%, and a specificity of 91.6%, lower than the lab-reported performance values (AUROC: 0.955, sensitivity: 92.5%, specificity: 98.5%). The corresponding gaps in sensitivity and specificity were 12.9% and 6.9%, respectively. The precision and *F*_1_-score at the image level were 38.6% and 52.0%, respectively. Evaluation at the individual level yielded 2062 (0.9%) FN, 33,878 (14.2%) FP, 186,648 (78.5%) TN, and 15,236 (6.4%) TP images. The sensitivity, specificity, and accuracy for identifying individuals with referable DR were 88.1%, 84.6%, and 84.9%, respectively. The precision and *F*_1_-score at the individual level were 31.0% and 45.9%, respectively. Subgroup analysis for each DR grade indicated label discrepancies between the DL algorithm and human labels, mainly accumulated in R1, R2, and R3a (Figure S2 in Multimedia Appendix 1).

**Table 1:** Grading results of the DL^a^ algorithm and the ground truth in identifying the presence of referable DR^b,c^.

Performance	Images (N=736,083)		Participants (N=237,824)	
	DL model	Human labels^d^	DL model	Human labels
Positive, n (%)	98,127 (13.3)	45,740 (6.2)	49,114 (20.7)	17,298 (7.3)
Negative, n (%)	666,055 (90.5)	690,343 (93.8)	188,710 (79.4)	220,526 (92.7)
TP^e^, n (%)	36,401 (5.0)	—^f^	15,236 (6.4)	—
FP^g^, n (%)	57,886 (7.9)	—	33,878 (14.2)	—
TN^h^, n (%)	632,457 (85.9)	—	186,648 (78.5)	—
FN^i^, n (%)	9,339 (1.3)	—	2,062 (0.9)	—
Accuracy, %	90.9	—	84.9	—
Precision, %	38.6	—	31.0	—
Sensitivity (recall), %	79.6	—	88.1	—
Specificity, %	91.6	—	84.6	—
*F*_1_-score, %	52.0	—	45.9	—

^a^DL: deep learning.

^b^DR: diabetic retinopathy.

^c^Referable DR was defined as preproliferative and proliferative DR.

^d^Human labels came from the final DR-grading results of human graders, based on the NHS DR-screening guidelines.

^e^TP: true positive.

^f^Not applicable.

^g^FP: false positive.

^h^TN: true negative.

^i^FN: false negative.

### Adjudication and Label Correction

Table 2 presents the adjudication results of extracted samples with inconsistent grading results between DL and human labels. After adjudication, 560 (63.6%) of 880 images labeled positive were deemed negative, leading to a mean estimated FN error rate of 63.6% (SD 1.6%, 95% CI 60.4%-66.8%). However, 140 (5.2%) of 2710 images labeled negative were deemed positive, with a mean estimated FP error rate of 5.2% (SD 0.4%, 95% CI 4.4%-6.1%). The adjudication at the individual level yielded a similar finding (FN error rate: mean 65.3%, SD 3.4%, 95% CI 58.2%-72.0%; FP error rate: mean 5.1%, SD 0.5%, 95% CI 4.1%-6.3%). Detailed classifications are listed in Table S2 in Multimedia Appendix 1.

**Table 2:** Adjudicated results of sample images.

Sample adjudication	Image level			Participant level	
	Images labeled positive (n=880)	Images labeled negative (n=2710)	Images labeled positive (n=195)		Images labeled negative (n=1657)
Erroneous labels	560 (63.6%)	140 (5.2%)	128 (65.3%)		85 (5.1%)
Accurate labels	320 (36.4%)	2570 (94.8%)	68 (34.7%)		1572 (94.9%)

**Figure 2:**
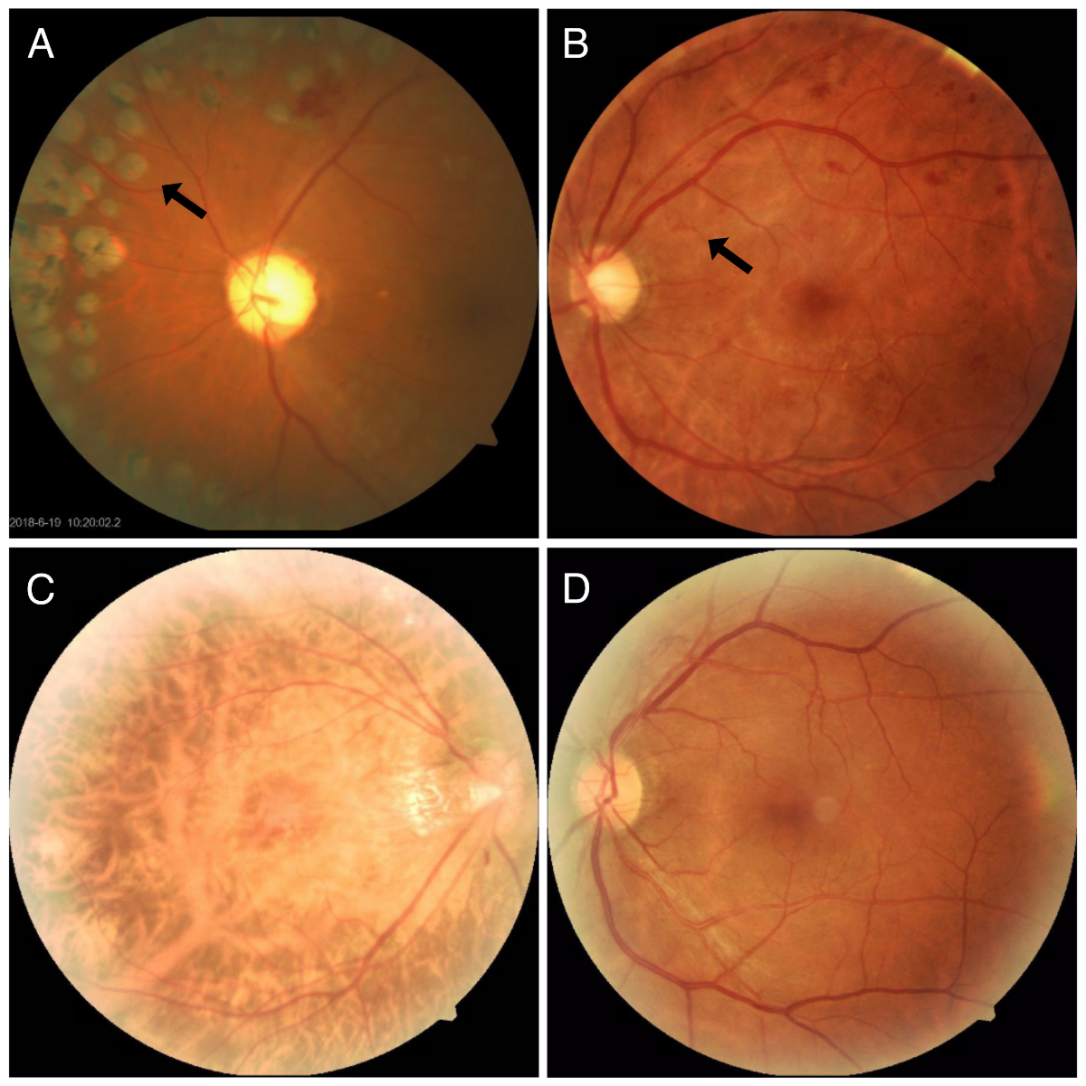
Typical images with human label errors before correction.

As shown in Table 3, the top reasons for misclassifying R0 images as referable DR images in precorrection labels included high myopia (n=106, 28.5%), a normal-appearing fundus (n=82, 22%), artifacts (n=40, 10.8%), and age-related macular degeneration (AMD; n=37, 10%). The predominant reasons for misclassified referable DR cases in precorrection labels included undetected laser spots (n=56, 41.8%), potential venous anomalies (n=25, 18.7%), and intraretinal microvascular anomalies (IRMAs; n=22, 16.4%). Typical images with erroneous labels are presented in Figure 2: Figures 2A and 2B show referable DR images misclassified as negative in human labels, with laser spots and retinal neovascularization, while Figures 2C and 2D show nonreferable DR images misclassified as positive in labels, probably due to a high-myopia fundus and artifacts.

**Table 3:** Potential reasons for errors^a^ in precorrection labels.

Probable reasons		Count, n (%)
**Images misclassified as positive**		
	High myopia	106 (28.5)
	Normal-appearing fundus	82 (22.0)
	Artifacts	40 (10.8)
	AMD^b^	37 (10.0)
	Underexposure	33 (8.9)
	Other retinopathies	20 (5.4)
	Poor quality	11 (3.0)
	Hypertensive retinopathy	8 (2.2)
	Media opacity	6 (1.6)
**Images m** **isclassified as negative**		
	Laser spots	56 (41.8)
	Venous anomalies	25 (18.7)
	IRMAs^c^	22 (16.4)
	Neovascularization	14 (10.5)
	Multiple blot hemorrhages	11 (8.2)
	Fibrous membrane	3 (2.2)
	Vitreous hemorrhage	1 (0.8)

^a^Errors in precorrection labels were estimated against the results of the adjudication. Images misclassified as positive represented those graded as referable diabetic retinopathy (DR) in the labels but turned out to be adjudicated as R0; images misclassified as negative represented those graded as nonreferable DR in the labels but turned out to be referable DR after adjudication.

^b^AMD: age-related macular degeneration.

^c^IRMA: intraretinal microvascular anomaly.

Based on the FN and FP error rates in the sample, we assumed that approximately 8933 images (from 3084 individuals) were misclassified in the precorrection labels in the entire data set, resulting in a mean estimated image error rate of 1.2% (SD 0.01%, 95% CI 1.1%-1.3%; individual level: mean 1.3%, SD 0.02%, 95% CI 1.1%-1.5%). The postcorrection labels of the entire data set were estimated to include 42,787 truly positive images and 693,296 truly negative images (from n=17,689, 7.4%, referable DR participants and n=220,135, 92.6%, nonreferable DR participants, respectively).

### Postcorrection Model Performance

**Figure 3:**
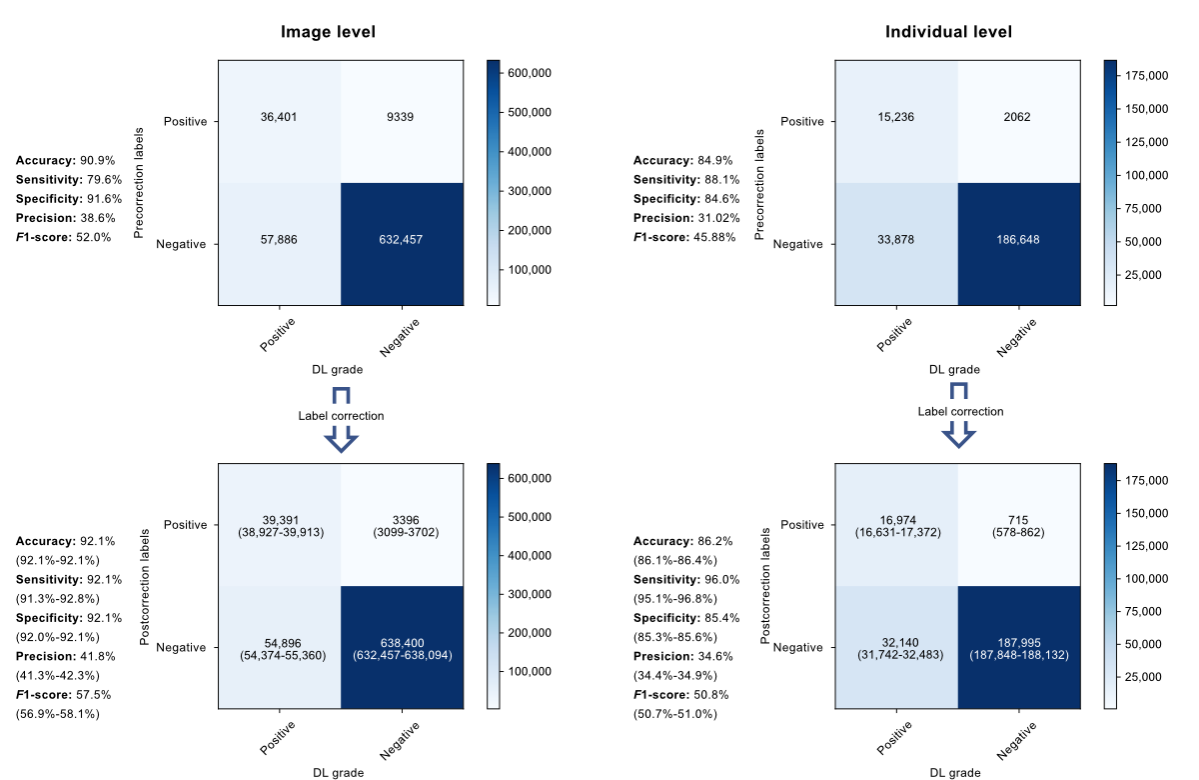
Performance of the DL model before and after label correction. DL: deep learning.

The confusion matrixes in Figure 3 show the comparison of DL model performance before and after label correction. After correction at the image level, sensitivity significantly improved by 12.5% (from 79.6% to 92.1%, *P*<.001), and the corresponding improvement in specificity and accuracy was 0.5% (from 91.6% to 92.1%, *P*<.001) and 1.2% (from 90.9% to 92.1%, *P*<.001), respectively. Similarly, postcorrection results at the individual level showed a 7.9% increase in sensitivity (from 88.1% to 96%, *P*<.001), a 0.8% increase in specificity (from 84.6% to 85.4%, *P*<.001), and a 1.3% increase in accuracy (from 84.9% to 86.2%, *P*<.001). The lab/real-world performance gap was significantly smaller after label correction, with a significantly reduced sensitivity gap from 12.9% to 0.4% (*P*<.001), as well as a reduced specificity gap from 6.9% to 6.4% (*P*<.001).

## Discussion

### Principal Findings

Based on a nationwide DR-screening program, we identified a significant gap between the lab-reported and real-world performance of a DL-based DR-screening model, which could be largely reduced by checking and correcting the errors in human labels. These results highlight the significance of acknowledging potential inaccuracies in human labels, which is often regarded as the gold standard. In this study, we proposed an approach of sampling adjudication to address this issue for a large data set. By evaluating the error rate within sampled subsets, we extrapolated and approximated the accurate distribution of labels across the entire data set. This approach enables a more precise evaluation of AI performance in real-world practice by providing estimates that consider and mitigate the impact of label errors.

Currently, there are 2 predominant approaches to generate a manually labeled gold standard in image-based real-world investigations [6-8,21-28]. The first approach involves a 2-stage grading process. In this process, 2 primary graders review all images, and a senior grader intervenes to make a final decision in the case of a disagreement between the primary graders [21]. The second method is a 1-stage grading process, wherein a single grader reviews all images to obtain human labels. In some instances, the 1-stage grading process might need additional verification for positive cases or a subsample of images [6]. Although accuracy may be somewhat compromised, the 1-stage grading method has advantages in terms of efficiency, convenience, time-effectiveness, and cost-effectiveness. Therefore, 1-stage grading is more commonly used in real-world practice, such as in the case of the Lifeline Express in this study. However, regardless of which grading method is used, we recommend double-checking human labels, even in a subsample of the data, before assessing the performance of the DL model in the real world.

Despite increasing endeavors aimed at enhancing the accuracy of human labels through measures such as standardized training for graders, the issue of mislabeling remains inevitable. Even if human graders make just 1 mistake in 100 cases, such as misclassifying different retinal conditions as DR or missing laser scars on the peripheral retina, it affects the gold standard. These small flaws can significantly influence the development and evaluation of AI algorithms. However, the amount of research available that evaluates the accuracy of manual grading outputs in DR screening is limited. In one study, 7379 images collected from 735 individuals were used to assess the performance of manual grading, which achieved a sensitivity of 82.2% and a specificity of 84.4% [7]. In another study involving 2384 images collected from 1208 participants in a nationwide DR-screening program in Thailand, the grading performance of human graders was deemed relatively reliable (accuracy: 93.5%, sensitivity: 84.8%, specificity: 95.5%) [28]. Moreover, Krause et al [29] compared DR-grading outcomes of a majority decision made by 3 ophthalmologists with the standard reference of adjudication by retinal specialists [29]. A discrepancy rate of around 0.8% can be drawn for identifying vision-threatening DR from their conclusion. These previous studies, in conjunction with our findings, support the need to estimate the error rate associated with manual grading in various real-world DR-screening settings. These estimates range from approximately 1% to 7%. Variations in the DR prevalence in the target population, grading workflow, and grader certifications may serve as contributing factors.

The label error rate in our study was around 1%, primarily attributed to the misclassification of nonreferable cases as referable DR. This is not anticipated to impede prompt referral and intervention for patients truly requiring medical attention. However, even such a small error rate can largely deteriorate the performance of DL algorithms in the real world. To address this concern, 1 previously proposed solution is to establish a rigorous reference standard during the human labeling process—for example, incorporating adjudication consensus from retinal specialists, rather than relying on major decisions made by multiple graders [29]. However, implementing a rigorous grading workflow in large-scale real-world screening practices can be time-consuming and resource intensive. Additionally, this process cannot be executed after study completion. Our study introduced an optimization strategy based on sample adjudication and label correction. Importantly, this strategy does not require modifications to the grading workflow or retraining of the DL model. Despite addressing only around 1% of label errors in the entire data set, this optimization strategy has been shown to bring about 12% enhancement in the AI model’s sensitivity.

The label correction strategy in our study can be considered a post hoc quality control procedure. It has been reported that label corrections for model tuning could effectively enhance the performance of AI algorithms [29]. Our findings further support the potential beneficial effect of adding this quality control procedure in existing and future DR-screening programs, especially when a large gap is observed between the real-world and lab-reported performance of the AI algorithm. However, implementation of this strategy in real-world practice may present challenges. Even if only a small subsample of images is selected for review, this would increase the workload of ophthalmologists, necessitating additional time and resources. Based on our study, we recommend that sampling approximately 5% of images with disagreement is reasonable for post hoc quality control. We recommend that future studies determine an appropriate sample size based on their expected error rate, resources, and other real-world considerations. We believe that this label correction strategy offers a standardized quality control pipeline across diverse image-based AI applications. Future investigations could explore the potential effect of this strategy in other AI-based models (eg, other diseases or imaging modalities) and other health care scenarios (eg, disease diagnosis or prognosis).

### Strengths

This study possesses several notable strengths. First, it was embedded in a nationwide DR-screening program, ensuring a large and robust data set comprising over 0.7 million fundus images collected from more than 0.2 million participants. This considerable scale enhanced the reliability of our findings. Second, to the best of our knowledge, this study represents a pioneering effort in evaluating the impact of label errors on the performance of a DR-grading DL algorithm. In addition, the proposal and validation of an effective optimization strategy in this study contribute significantly to the understanding and advancement of DL implementation in real-world DR screening.

### Limitations

This study has some limitations. In our evaluation, images with identical outputs between DL and labels (TP and TN cases) were considered correctly classified, without undergoing further adjudication. In smaller-scale trials, we adjudicated a limited subset of TP (200 images) and TN (1000 images) cases. These assessments revealed a minimal error rate in human labeling, at 2.5% for TP and 0.1% for TN (further details in Table S3 in Multimedia Appendix 1). Given the substantial quantity of TP and TN images within our data set, refraining from label correction in these subsets would unlikely cause a significant alteration in the estimated performance of the DL model. Additionally, during the adjudication process, we conducted random sampling instead of reviewing all images with discrepancies. To ensure the representativity of the random sample, we performed multiple samplings with varying sample sizes and validated consistent distributions of the obtained results. Moreover, the Lifeline Express program exclusively recruited Chinese participants. It is imperative to conduct additional validation studies encompassing diverse ethnicities to corroborate our findings. Lastly, our analysis focused solely on a single DR-screening model. Nevertheless, it is reasonable to infer that our conclusions are applicable to alternative models used in the screening of other ocular diseases. Therefore, further investigations are warranted to substantiate this discovery.

### Conclusion

In conclusion, we found that even a small percentage of label errors could have a substantial impact on the performance of DL algorithms in real-world DR screening. Attention should be paid to minimizing and correcting label errors in future studies and implementations of AI-based DR-screening models in the real world.

## Appendix

Multimedia Appendix 1 Supplementary tables and figures.

## Abbreviations

AI: artificial intelligence
AUROC: area under the receiver operating characteristic curve
DL: deep learning
DR: diabetic retinopathy
FN: false negative
FP: false positive
NHS: National Health Service
TN: true negative
TP: true positive
