# Supplementary material for: Impact of Gold-Standard Label Errors on Evaluating Performance of Deep Learning Models in Diabetic Retinopathy Screening: Nationwide Real-World Validation Study
Source: J Med Internet Res. 2024 Aug 14;26:e52506. doi: 10.2196/52506 (PMC11358665; doi:10.2196/52506)
Supplement: Multimedia Appendix 1 [file jmir_v26i1e52506_app1.docx]

### Multimedia Appendix 1

| **Table S1.** Criteria for quality control and DR grading according to the NHS guidelines. | |
| --- | --- |
| **Classification** | **Presence of Clinical Features** |
| **Quality Control** | |
| Poor quality | Any criterion of the following: |
|  | 1) Vessels within 1 DD of the optic disc margin or macular fovea cannot be identified |
|  | 2) >= 50% of the area is obscured |
| Poor location | Central of the image deviates from the optic disc or the macular fovea more than 2 DD |
| **DR grading** | |
| R0 | Does not meet any of the following criteria |
| R1 | R1.1) Microaneurysm |
|  | R1.2) Retinal hemorrhage |
|  | R1.3) Hard Exudate |
|  | R1.4) Cotton-wool spot |
| R2 | R2.1) Venous loops |
|  | R2.2) Venous beading |
|  | R2.3) Venous reduplication |
|  | R2.4) Intraretinal microvascular abnormality |
|  | R2.5) Multiple blot hemorrhages |
| R3s (stable) | R3s.1) Evidence for peripheral retinal photocoagulation and a stable condition after treatment |
|  | R3s.2) Stable fibrous proliferation with or without tractional retinal detachment |
| R3a (active) | R3a.1) Neovascularization of the disc or neovascularization elsewhere in the retina |
|  | R3a.2) Preretinal hemorrhage or vitreous hemorrhage |
|  | R3a.3) Active fibrous proliferation with traction over the retina |
|  | R3a.4) Tractional retinal detachment |
| DR = diabetic retinopathy; NHS = National Health Service; DD = disc diameter. | |
| Images of poor quality or poor location would be classified as ungradable, otherwise as gradable. Referable DR was defined as R2 and grades above. | |

| **Table S2.** Detailed adjudicated findings and distribution of DR categories in sample images. | | |
| --- | --- | --- |
| **Adjudicated results** | **False negative sample** | **False positive sample** |
| **Images** |  |  |
| No. of randomly sample | 934 | 2,894 |
| Poor quality | 54 (5.78%) | 184 (6.36%) |
| R0 | 380 (41.3%) | 960 (33.6%) |
| R1 | 179 (19.4%) | 1,610 (56.4%) |
| R2 | 49 (5.32%) | 66 (2.31%) |
| R3s | 166 (18.0%) | 43 (1.51%) |
| R3a | 106 (11.5%) | 31 (1.09%) |
| Nonreferable DR | 560 (60.8%) | 2,570 (90.0%) |
| Referable DR | 320 (34.7%) | 140 (4.90%) |
| Error Rate, Mean (95%CI) | 63.6% (60.4%-66.8%) | 5.17% (4.36%-6.07%) |
| **Participants** |  |  |
| No. of randomly sample | 206 (10%) | 1,694 (5%) |
| Poor quality | 10 (4.85%) | 37 (2.18%) |
| R0 | 59 (29.1%) | 464 (27.8%) |
| R1 | 69 (33.4%) | 1,108 (66.3%) |
| R2 | 11 (5.42%) | 32 (1.91%) |
| R3s | 34 (16.8%) | 29 (1.73%) |
| R3a | 23 (11.3%) | 24 (1.44%) |
| Nonreferable DR | 128 (63.1%) | 1,572 (94.02%) |
| Referable DR | 68 (33.5%) | 85 (5.08%) |
| Error Rate, Mean (95%CI) | 65.3% (58.2%-72.0%) | 5.13% (4.12%-6.30%) |

### DR = diabetic retinopathy; CI = confidential interval.


| **Table S3.** Label errors for TP and TN images in sampling adjudication. | |
| --- | --- |
| **Adjudication results** | **Label positive (n=200)** |
| Ungradable | 3 |
| Moderate NPDR | 3 |
| BRVO | 1 |
| Hypertensive retinopathy | 1 |
|  | **Label negative (n=1000)** |
| Ungradable | 25 |
| PDR with pre-retinal hemorrhage | 1 |

TP = true positive, TN = true negative, NPDR = non-proliferative diabetic retinopathy, BRVO = branch retinal vein occlusion.

We performed a small-scale sampling and adjudication for TP and TN images. A total of 200 TP images (AI and human graders both gave positive results) and 1000 TN images (AI and human graders both gave negative results) were randomly sampled and review by ophthalmologists. The adjudication results found 5 mistakes in positive human labels and 1 mistake in negative human labels, leading to human label error rates of 2.5% in TP subsets and 0.1% in TN subsets. When combining these error rates with the label correction procedure in the main text, the TP, TN, FP, and FN counts in the entire dataset would be estimated as 38,481, 637,768, 55,806, and 4028, respectively. The estimated performance of the DL model postcorrection would be 91.9% accuracy, 90.5% sensitivity, and 92.0% specificity.

### Figure S1. Distribution of error rate in human labels by gradual sampling of false negative images.


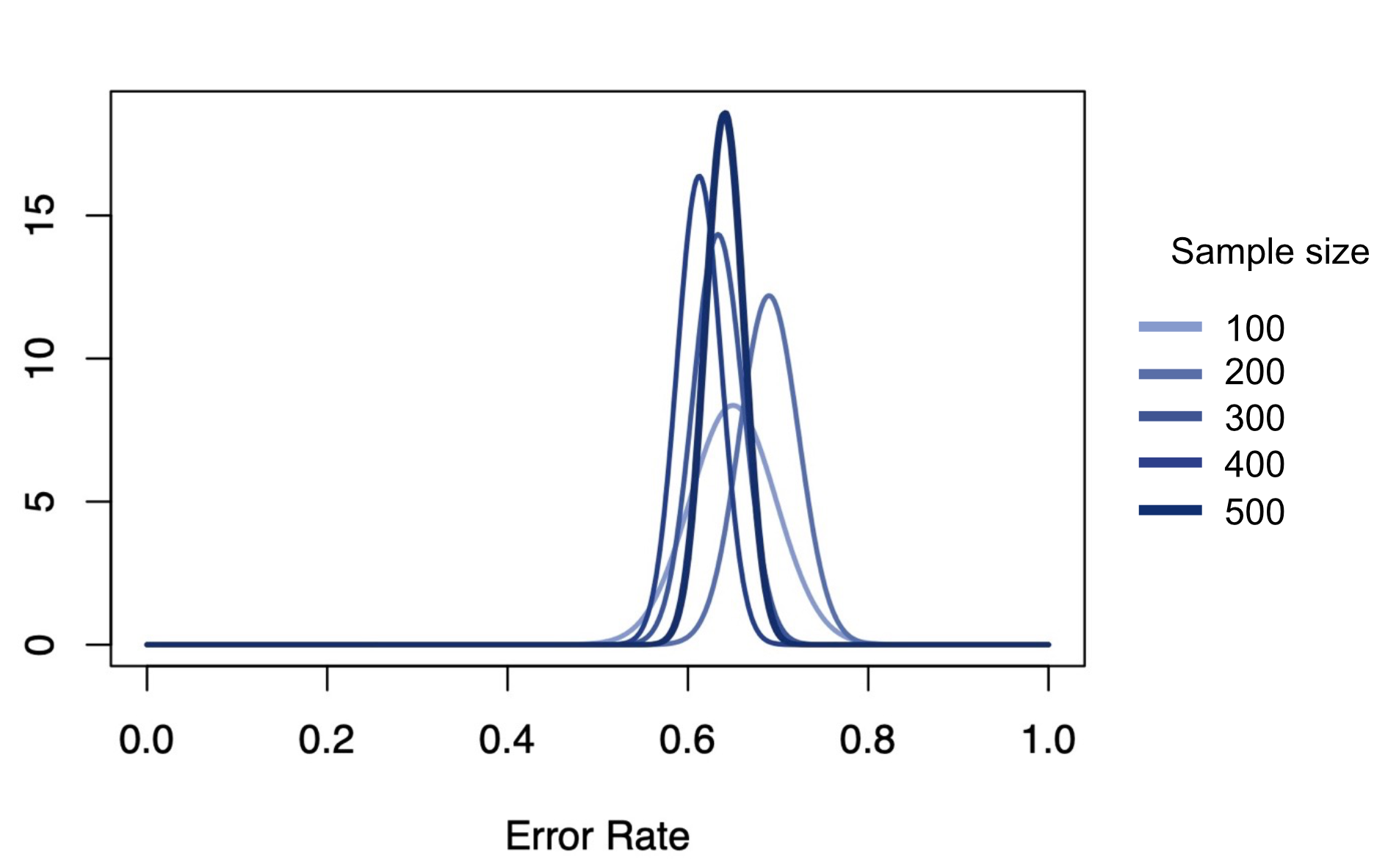


FN = false negative, DR = diabetic retinopathy.

To exam an even distribution of extracted random samples, we applied a six-times gradual sampling of false negative (FN) images with a sample size of 100, 200, 300, 400, 500, and 500, respectively. In each sample, around 61.3%-69.0% of images were found having error labels during the adjudication review. No significant difference was found among estimations of error rate (p=.61). Therefore, it can be inferred that even a subset as small as 100 images can effectively represent the label distribution within the entire data set.

**Figure S2.** Subgroup analysis on prediction of the DL algorithm detecting different grades of DR at image level before label correction.


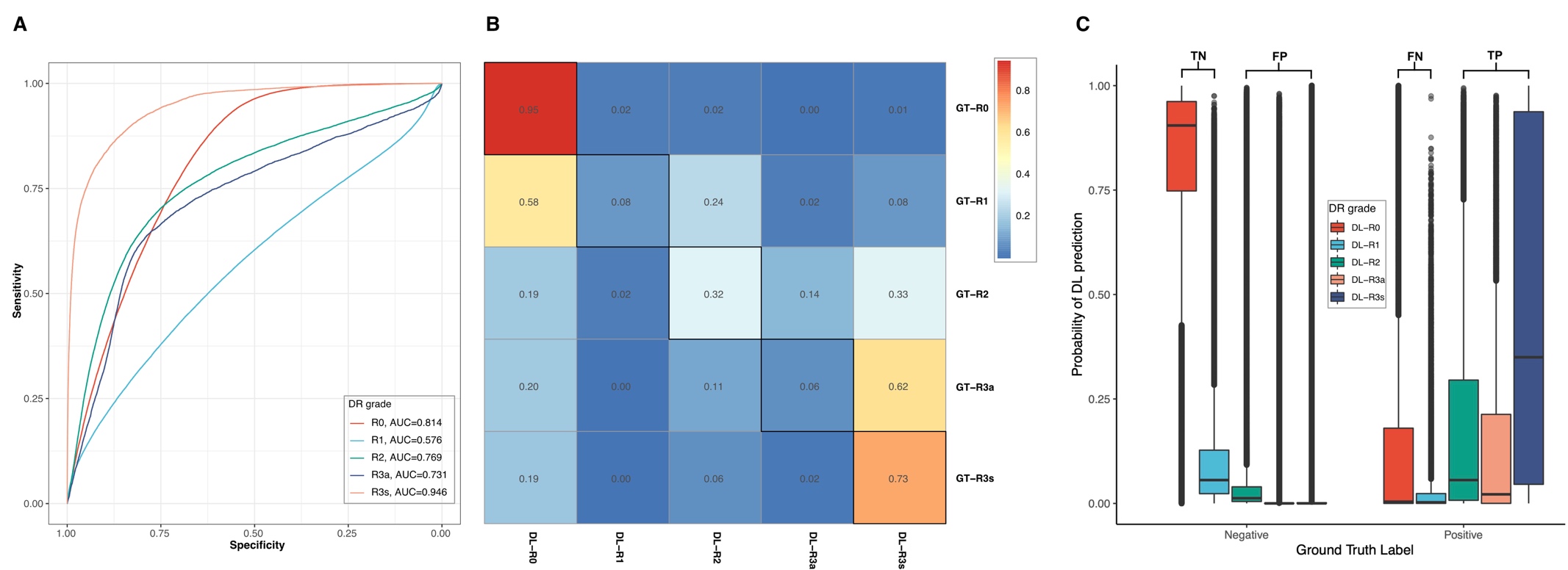


ROC = receiver operating characteristic; DL = deep learning; DR = diabetic retinopathy; GT = ground truth; AUC = area under the curve. **A)** The ROC curve and corresponding AUC for each DR grade, as well as the general performance of the DL algorithm. **B)** Distribution of DL and human labels. For example, the rectangle located at the top left represents that 95% of images of the R0 grade in labels were classified as R0 by the DL. Rectangles with black edges indicate consistent grading results between the DL and labels. **C)** Distribution of probability score predicted by the DL in label-positive and negative cases. A high probability score in the R0 grade means a high tendency of DL to make a prediction of R0 as compared to other DR grades in label-negative images.
